# Independent evolution of cutaneous lymphoma subclones in different microenvironments of the skin

**DOI:** 10.1038/s41598-020-72459-9

**Published:** 2020-09-23

**Authors:** Aishwarya Iyer, Dylan Hennessey, Sandra O’Keefe, Jordan Patterson, Weiwei Wang, Gane Ka-Shu Wong, Robert Gniadecki

**Affiliations:** 1 Department of Medicine, University of Alberta, Edmonton, AB T6G 2G3 Canada; 2 Division of Dermatology, University of Alberta, 8-112 Clinical Sciences Building, 11350-83 Avenue, Edmonton, AB Canada; 3 Department of Oncology, Cross Cancer Institute, University of Alberta, Edmonton, AB Canada; 4 Department of Biological Sciences, University of Alberta, Edmonton, AB Canada; 5 Geneis, Beijing, China; 6 Department of Dermatology, Bispebjerg Hospital, University of Copenhagen, Copenhagen, Denmark

**Keywords:** Cancer genomics, Tumour heterogeneity

## Abstract

Mycosis fungoides (MF) is the most common cutaneous T-cell lymphoma. Lesions of MF are formed by hematogenous seeding the skin with polyclonal (clonotypically diverse) neoplastic T-cells which accumulate numerous mutations and display a high degree of mutational, intratumoral heterogeneity (ITH). A characteristic but poorly studied feature of MF is epidermotropism, the tendency to infiltrate skin epithelial layer (epidermis) in addition to the vascularized dermis. By sequencing the exomes of the microdissected clusters of lymphoma cells from the epidermis and the dermis, we found that those microenvironments comprised different malignant clonotypes. Subclonal structure witnessed the independent mutational evolution in the epidermis and dermis. Thus, the epidermal involvement in MF could not be explained by gradual infiltration from the dermis but was caused by a separate seeding process followed by a quasi-neutral, branched evolution. In conclusion, tissue microenvironments shape the subclonal architecture in MF leading to “ecological heterogeneity” which contributes to the total ITH. Since ITH adversely affects cancer prognosis, targeting the microenvironment may present therapeutic opportunities in MF and other cancers.

## Introduction

Mycosis fungoides (MF) is one of the most common diseases in the realm of extranodal T-cell lymphomas^1^. It is a skin-tropic lymphoid neoplasm that initially presents as scaly, erythematous patches and plaques, which may progress to tumours and disseminate to lymph nodes and other organs^2–4^.

ITH has recently emerged as an important characteristic of solid and hematopoietic malignancies^5^. Although mutations in few driver genes may be sufficient to initiate tumorigenesis, it is now evident that the progression depends on the accumulation of multiple mutations to promote expansion and invasion of the primary niche and surrounding tissues^6,7^. Mutations occur randomly in malignant cells within the tumour, leading to the emergence of multiple subclones. ITH allows cancer to withstand selection pressure from the microenvironment and therapies by promoting the expansion of subclones harboring mutations advantageous to these cells^6^.

The generation of ITH is usually viewed as an evolutionary process with a single transformed cell as a starting point. This cell proliferates and branches into phylogenetically related subclones (Supplementary Fig. S1) that infiltrate the tissue. Although this model may be applicable to cancers that grow expansively as single tumours, this is not necessarily true for all malignancies. Many cancers comprise the entire ecosystem of primary and metastatic lesions that are physically separated from each other. It has been shown that in such situations, tumour heterogeneity may be augmented by cross-seeding by circulating, genetically diverse cancer subclones, for example, cancer self-seeding by the cells from the metastatic lesion re-entering the primary tumour^8^. We hypothesized that a similar mechanism may operate at the microscopic scale for primary cancers, where different compartments within an organ can be colonized by different cancer subclones. Independent seeding of different microscopic compartments within the same organ would increase the heterogeneity of the entire lesion beyond what would have been possible by a continuous evolution from only one ancestral clone (Supplementary Fig. S1).

MF provides a convenient model to test this hypothesis. The skin has a simple layered structure comprising ectodermal derived epidermis and the mesodermal dermis. Both layers can be occupied by cancer cells in MF. The histopathology of MF reveals disconnected areas of malignant cell clusters in the dermis and the epidermis. Dermal infiltrate is usually perivascular or diffuse whereas lymphoma foci in the epidermis form well-demarcated clusters of cells known as Pautrier abscesses **(**Supplementary Fig. S2). Pautrier microabscesses are a characteristic feature of MF and are present in approximately 20% of all biopsies^9,10^. Unlike the dermal perivascular infiltrates that comprise a significant proportion of reactive cells, Pautrier abscesses are believed to contain predominantly cancer cells with a minor admixture of apoptotic Langerhans cells and eosinophils^11^. The initial points of entry of malignant cells are the capillaries in the upper (papillary) dermis. Therefore, Pautrier microabscesses are a manifestation of the infiltrative growth in MF by which “epidermotropic” subclones migrate from the dermis to the epidermis.

We and others have recently studied the heterogeneity of MF on the genomic, transcriptomic and cellular levels^12–15^. In contrast to previous views considering MF as a relatively simple, monoclonal lymphoproliferation derived from a mature T-cell, we showed that MF comprises multiple mature T-cell clones which undergo branched evolution producing generations of cancer subclones^16,17^. Interestingly, there seems to be very little competition between different subclones and the disease progression is associated with an increase in subclonal diversity rather than a selection of the fittest subclones. We have therefore asked whether different microcompartments in the skin (epidermis vs dermis) play a role in the generation of ITH in MF. We found that Pautrier microabscesses do not comprise a subpopulation of the dermal malignant cells emigrating to the epidermis, but that they originate independently from distinct seeding events and undergo autonomous, branched evolution.

## Results

### Clonotypic diversity of malignant T-cells in epidermal and dermal niches in the skin

Since the epidermis is not vascularized, the intraepidermal neoplastic cells of Pautrier microabscesses must necessarily originate from the cells that initially enter the papillary dermis. Therefore, Pautrier microabscesses are assumed to represent a fraction of the dermal cells that acquired an ability to survive and proliferate in the epidermis. To examine this hypothesis we microdissected atypical cells from both layers (epidermal and dermal) of the skin in 7 MF patients (Supplementary Table S1) and analysed their clonotypic composition by comparing their T-cell receptor β (TCRβ) repertoires. We used the previously described methodology where the CDR3 sequences of the rearranged *TCRB* genes are detected by bioinformatic analysis of WES data^15^. Since *TCRB* locus is rearranged only on one chromosome (allelic exclusion) at the stage of the double-positive thymocyte, the unique CDR3 sequences constitute a molecular barcode identifying a single clone of the T-cell^18^.

**Figure 1:**
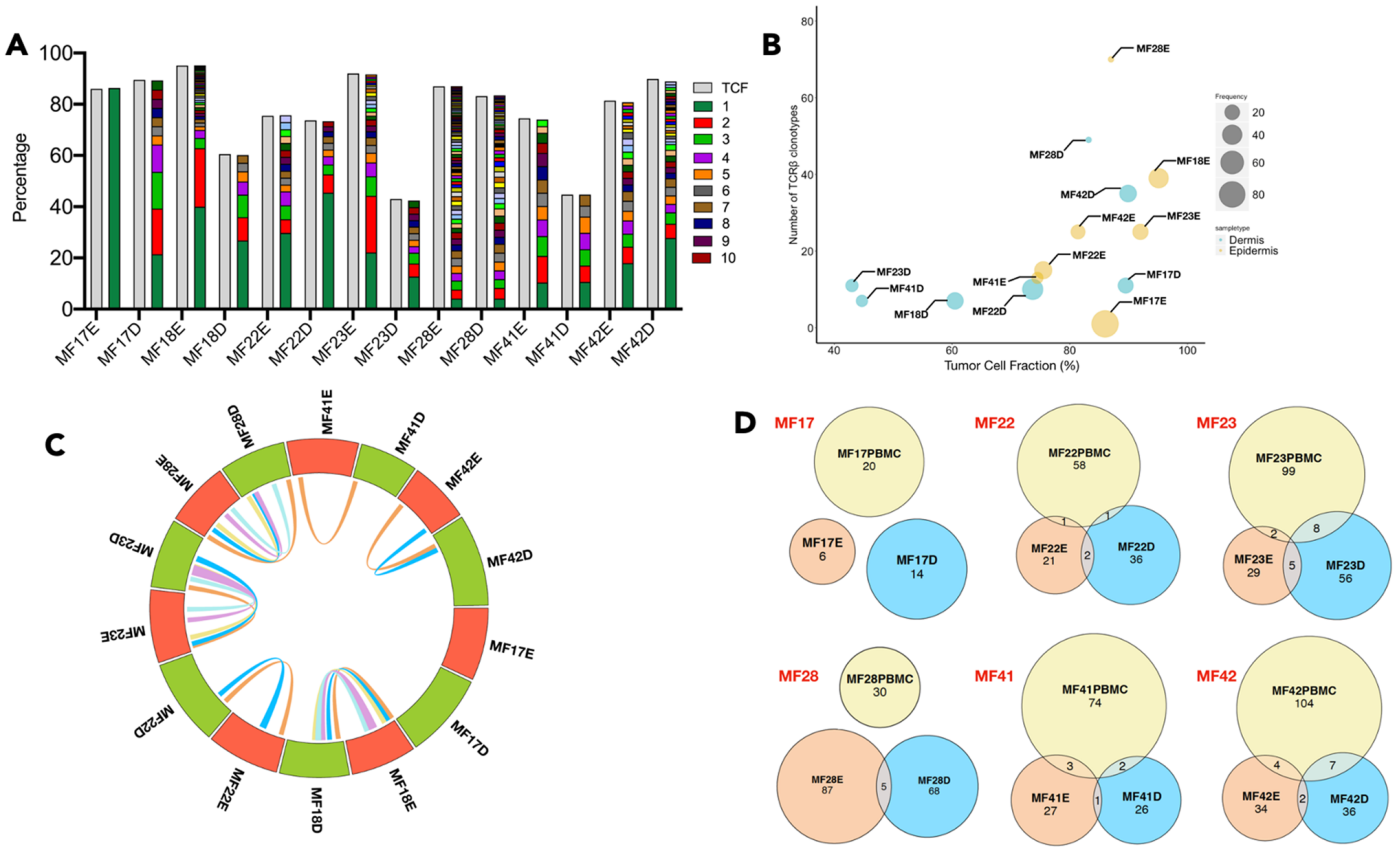
Clonotypic heterogeneity and tumour cell seeding of the skin microenvironment in MF. Percentage of Tumor cell fraction (TCF) and relative frequency of TCRβ clonotype sequences for cells isolated from different skin layer (epidermis and dermis) was calculated and plotted as a bar graph (**A**) The green and brown colour indicate the first and the 10th most frequent TCRβ clonotype in the sample. Gray colour indicates the tumour cell fraction (TCF). (**B**) Bubble plot presenting the correlation between TCF and the number of neoplastic TCRβ clonotypes in cells from epidermis and dermis of each sample. The size of the bubble is equivalent to the relative frequency of the most frequent TCRβ clonotype in the sample. (**C**) Circos plot indicates the frequency of TCRβ clonotype for cells isolated from epidermis and dermis of each sample. The connecting lines inside indicate the number of overlapping TCRβ clonotypes between the two regions of the same sample. E-Epidermis; D-Dermis. (**D**) Venn diagram indicating the number of identical TCRβ clonotypes between the epidermis, dermis and the circulating blood in samples MF17, MF22, MF23, MF28, MF41 and MF42.

We identified malignant TCRβ clonotypes by matching their frequency to TCF of the samples which eliminated the possibility that the clonotypes were derived from inflammatory T-cells^15^. We noticed that epidermal samples had a higher TCRβ clonotype diversity in comparison to the dermis (median of 25 clonotypes (range 1–70) versus 11 clonotypes (range 7–49), respectively) (Fig. 1A,B). However, the number of shared clonotypes between epidermis and dermis was very low, from no shared clonotypes (sample MF17), 1 shared clonotype (MF41) to a maximum of 2–5 clonotypes (MF18, MF22, MF23, MF28 and MF42) (Fig. 1C). These results indicated that the pools of malignant T-cells in the epidermis and dermis are largely clonotypically unrelated that suggested that they originate from separate seeding events by different T-cell clones. Indeed, we observed that in 4 of 6 samples analyzed, T-cells from the epidermal and dermal compartments individually shared between 1–8 TCRβ clonotypes with those in the circulating blood (Fig. 1D), which represented a higher degree of overlap than seen for the epidermal and dermal compartments. Taken together, the epidermal and dermal compartments of the skin are likely to be seeded by different circulating malignant clones.

### Mutational diversity in neoplastic T-cells in epidermis and dermis

The substantial clonotypic discordance between the epidermal and dermal compartments prompted a question regarding differences and similarities in their mutational evolution. In our previous work, we characterized 75 putative driver mutations involved in the pathogenesis and progression of MF^16^. Similarities in the patterns of driver mutations between the epidermal and the dermal infiltrate would suggest parallel evolution in both compartments whereas lack of substantial overlap would indicate a neutral evolution.

**Figure 2:**
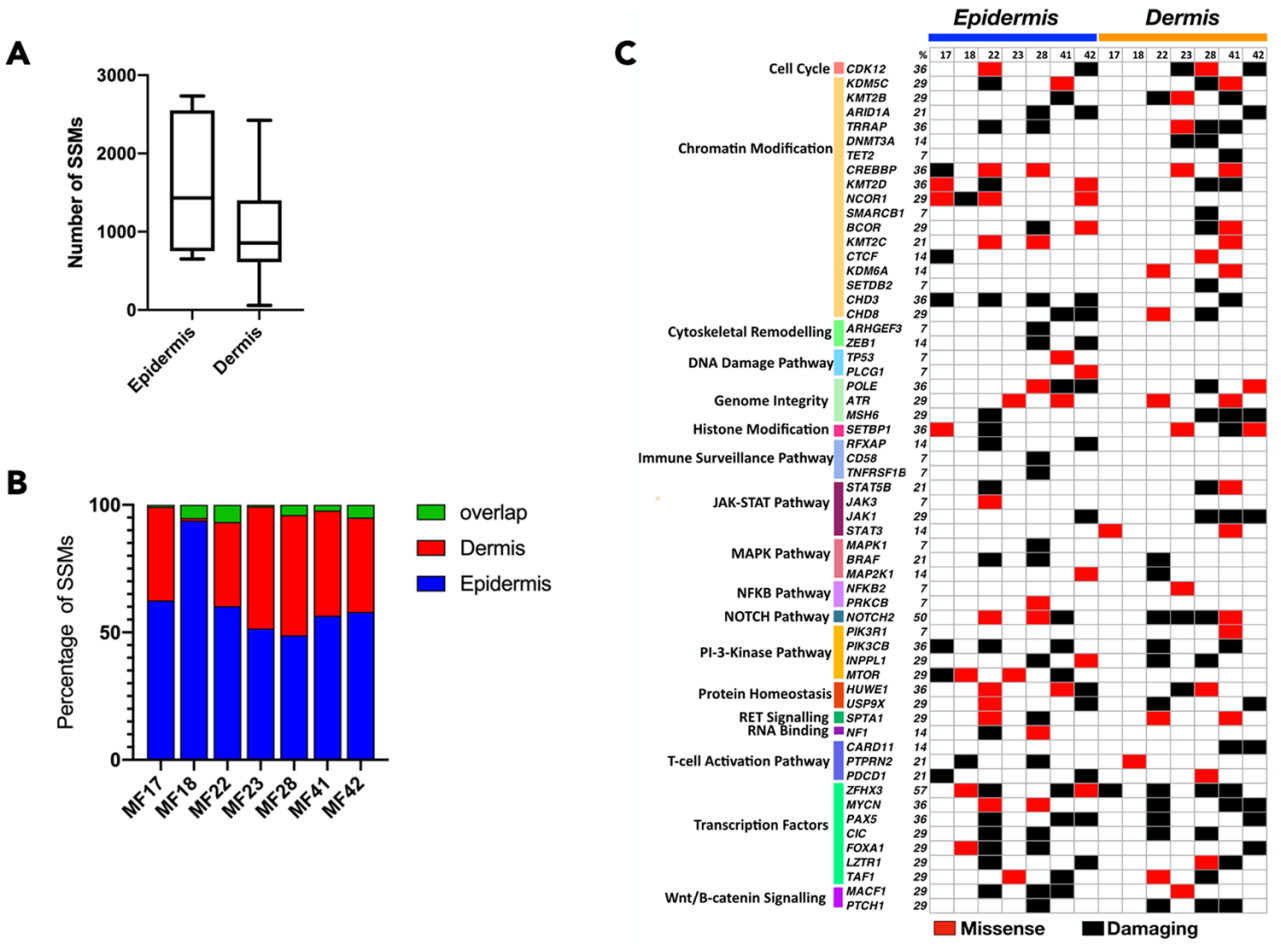
Mutational landscape of putative driver genes in anatomical layers of skin. Neoplastic T-cells isolated from epidermis and dermis were analyzed for somatic variants (SVs) in putative driver genes. (**A**) Number of non-synonymous SVs in neoplastic cells isolated from epidermis and dermis. Box and whisker plot showing 90th percentile respectively. (**B**) Bar graph represents the number of SSMs identified and the percent overlapping mutations between epidermis and dermis. (**C**) Mutations in 59 genes across 18 different pathways were identified. The mutations were classified as missense or damaging. Frameshift, insertion or deletion (< 6 bp), stop gain or lost are classified as damaging as these mutations are likely to be deleterious.

We identified a median of 856 non-synonymous mutations in cells from the dermal region and 1,431 non-synonymous mutations in cells from the epidermal region (Fig. 2A). The majority of the mutations (48–93%) were in the Pautrier microabscess fraction and the overlap between the compartments was less than 7% across all 7 samples (Fig. 2B). When driver genes were considered, 37 drivers were mutated in both epidermis and dermis, 13 genes (*NCOR1, ARHGEF3, ZEB1, TP53, PLCG1, RFXAP, CD58, TNFRSF1B, JAK3, MAPK1, PRKCB, MTOR* and *NF1*) were exclusively mutated in epidermis and 9 genes (*DNMT3A, TET2, SMARCB1, KDM6A, SETDB2, STAT3, NFKB2, NOTCH2* and *CARD11*) were mutated only in dermis (Fig. 2C). The mutations present only in the malignant T-cells of epidermis were in the genes involved in cytoskeletal remodelling, DNA damage and immune surveillance. However, the driver mutation profile in the epidermal and dermal fractions showed non-overlapping patterns arguing against parallel evolution. Thus, the data supported the model of independent mutational evolution of neoplastic cells in different skin microenvironments.

### Phylogenetic development of tumor T-cells in skin microenvironment

**Figure 3:**
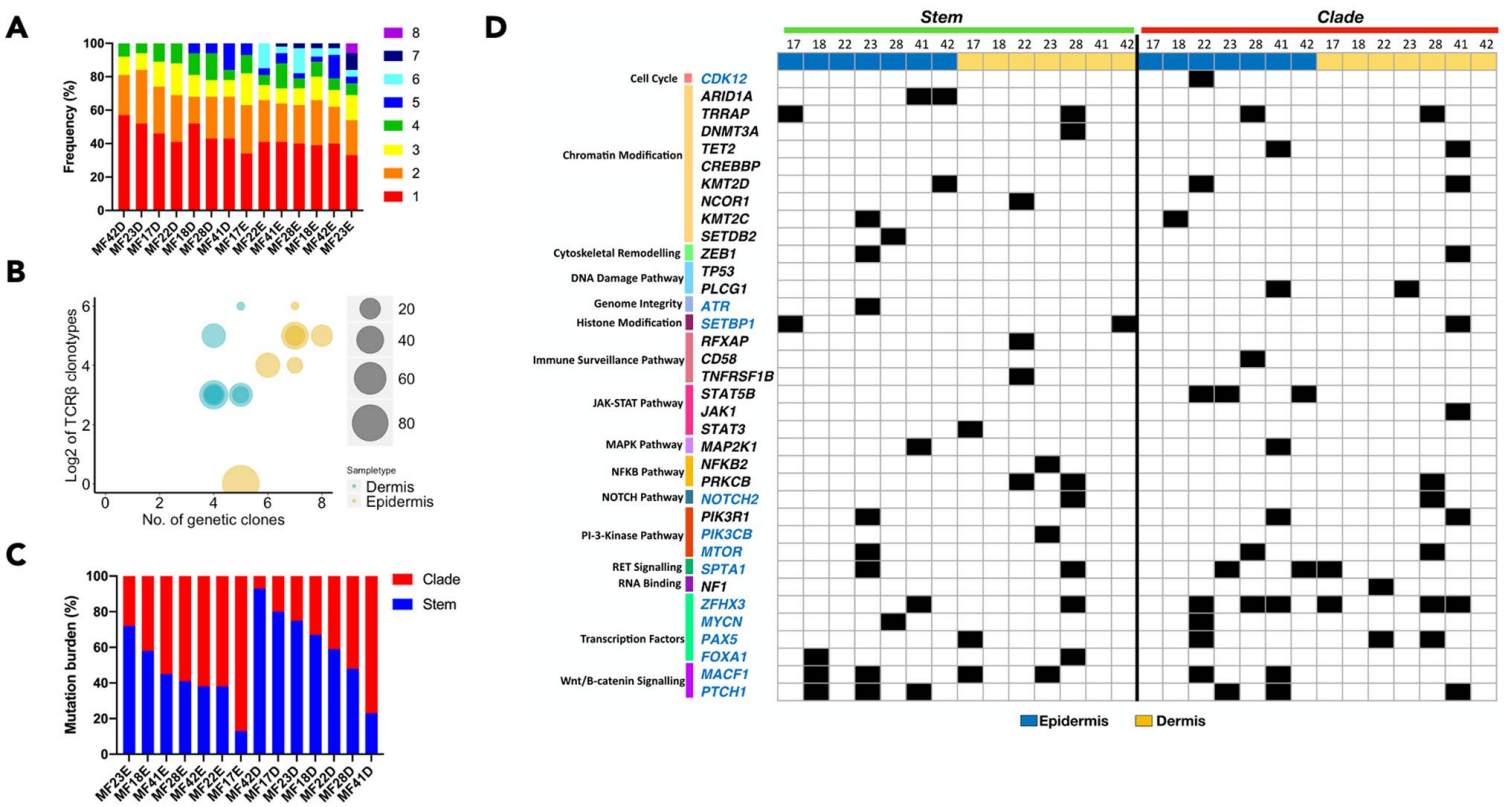
Evolutionary facets of the genetic clones in the skin microenvironment. Combined data from SVs and CNA for each sample was subjected to phylogenetic analysis to identify genetic subclones. (**A**) Rainbow graph representing the number and proportion of the subclones identified in each sample. (**B**) Bubble plot representing the correlation between the TCRβ clonotypes and the genetic subclones. The number of TCRβ clonotypes are represented as Log2 scale. (**C**) Phylogenetic trees are composed of stem and clades (also recognized as branches). Bar graph represents the percentage of all mutations in each section (stem and clade) of the phylogenetic tree. The blue and red colour represents the mutations in stem and clades respectively (**D**) Mutational landscape of the putative driver genes in the different sections of the phylogenetic tree for two layers of skin (epidermis and dermis). Function significance of the mutations include missense, frameshift, insertions, deletions, stop gain or loss and variant in 3′ and 5′ UTR. No colour indicates absence of mutation in the sample.

To further examine the phylogenetic relationships between the subclones in the epidermal and dermal compartments we adopted the previously described bioinformatic approach based on the analysis of the mutational pattern between cancer cells^16^. We found evidence of subclonal heterogeneity in all samples confirming previous findings of ITH in MF^16^. A slightly higher number of subclones were found in epidermal (5–8 subclones) versus the dermal layers (4–5 subclones) (Fig. 3A) reflecting the differences in clonotypic richness between those compartments (Fig. 3B). In the epidermal fraction, the mutational burden was mostly in the clades whereas the dermal fraction tended to have a higher proportion of clonal (stem) mutations (Fig. 3C). Thus, the number of subclones correlated with the proportion of subclonal mutations, as predicted for the neutral, branched evolution pattern^19^. We also analyzed driver gene mutations in the stem and clade population for the dermis and epidermis and found that mutations in *STAT5B* and *CDK12* where only present in clades in Pautrier microabscesses whereas *NOTCH2* and *PRKCB* mutations were only in the dermal fraction, either in the stem or clades (Fig. 3D).

**Figure 4:**
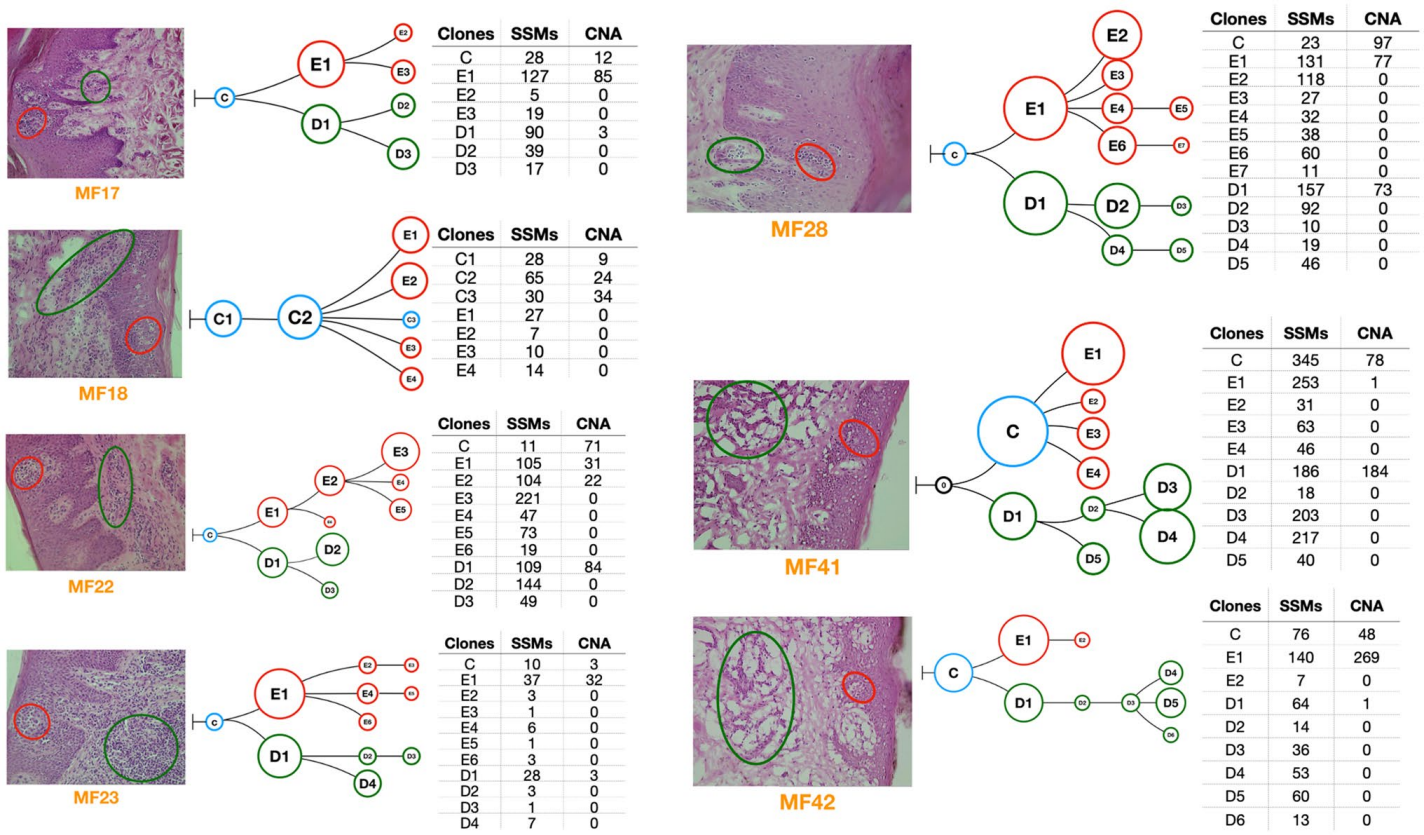
Phylogenetic analysis of the neoplastic T-cells in skin microenvironment. Genetic abnormalities (SVs and CNA) for neoplastic cells microdissected from epidermis and dermis were subjected to phylogenetic analysis. Each phylogenetic tree represents an individual patient sample. The blue circles indicate the common clone between the two skin layers. Red and green indicate the subclones in epidermis and dermis respectively. Black circles indicate absence of the common ancestral clone. The tables adjacent to each figure provide the number of SVs and CNA identified in each of the subclones in the phylogenetic tree.

To visualize how different subclones in the clades are related to each other, we reconstructed the phylogenetic trees. In one case (MF41) there was no common ancestor clone linking epidermal and dermal subclones. In other cases we detected 1–2 subclones forming the stem of the tree. All samples showed branched evolution of the subclones, with the epidermal and dermal clades clearly separated from each other (Fig. 4).

## Discussion

**Figure 5:**
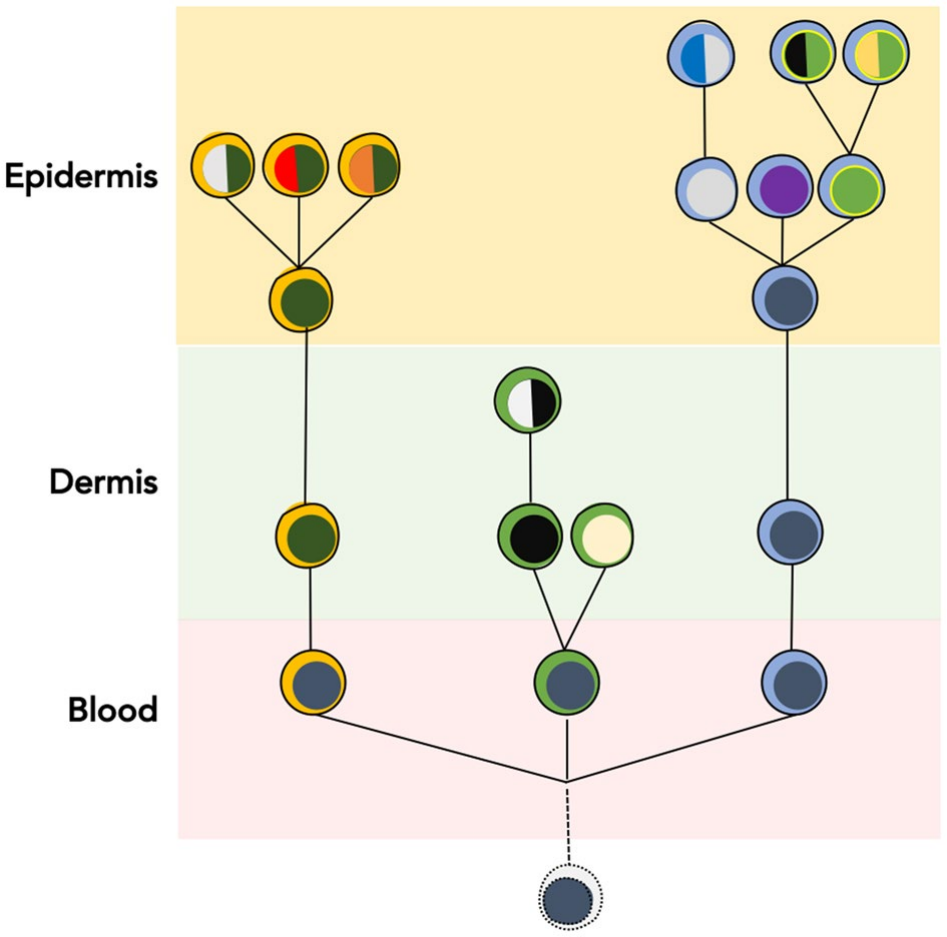
Generation of ecological heterogeneity in MF. Skin lesions of MF are initiated by circulating, clonotypically heterogeneous malignant T-cell clones (various clonotypes are highlighted by different colours of the “cytoplasm”). Upon entering the skin some clones remain in the dermis where they proliferate whereas others pass directly to the epidermis. Expanding clones accumulate mutations leading to emergence of genetically different malignant subclones (different colours of the “nucleus”). Solid lines symbolize the phylogenetic relationship between the generations of malignant cells and illustrate divergent, neutral evolution of the subclones. Based on data in this paper and our previous work^15,17,37,38^.

Tissue microenvironment has been recognized as a major factor that influences tumour cell morphology and function^20,21^, but the impact of the niche on ITH and mutational evolution is poorly understood and largely limited to metastasis^22,23^. Current results help to understand how the distinct microenvironments of the skin influence the evolution of MF, a primary cutaneous, extranodal T-cell lymphoma. Our previous research showed that MF is clonotypically and genetically diverse exhibiting a high degree of ITH. The main mechanism responsible for the heterogeneity is the hematogenous seeding of skin lesions by clonotypically diverse neoplastic T-cells. The finding that the epidermal and dermal layers of the skin comprise distinct malignant clonotypes allowed us to conclude here that those compartments had been colonized by different clones of cancer cells (in this context, we define the clone as a population of malignant T-cells that are derived from the common precursor cell and exhibit the same TCRβ clonotype). Thus, the lesion of MF does not develop via a gradual infiltration of the tissue by the expanding tumour, but by independent microinvasion events in which different niches in the skin are colonized independently by various T-cell clones (Fig. 5 and Supplementary Fig. S1A). Our conclusion was further confirmed by the finding that clonotypic diversity of the intraepidermal malignant cells exceeded the diversity found in the dermis. If the infiltration of the epidermis had been caused by some clones in the dermal infiltrate, the opposite phenomenon would have been found, i.e. higher number of malignant clonotypes in the dermis and a smaller number of epidermal clonotypes overlapping with the dermal clones. These findings reinforce and broaden the concept of epidermotropism in MF, which originally described the morphological impression of movement of malignant T-cells from the epidermis to the dermis. It seems that epidermotropism is a feature of early malignant T-cell clones which seed the epidermis more readily than the dermis.

We were also able to conclude that the existence of different skin niches colonized by cancer facilitates the development of mutational subclones and augments ITH. By analyzing the phylogenic trees of MF we found that cancer subclones seem to develop independently in each compartment via a quasi-neutral, branched evolutionary process. Similar patterns compatible with neutral evolution have previously been found in other solid neoplasms such as the lung or colorectal cancers^19,24^. Although it is tempting to speculate that evolving subclones in different compartments do not directly compete for the niche or the nutrients and develop independently, it is also possible that mutual competition/collaboration between clones (the so-called rock-paper-scissors type of interaction^25–27^) may contribute to ITH. Systematic, longitudinal sampling of evolving MF lesions might provide a good model for analysis of the mechanisms governing tumor evolution.

High ITH of the tumors has been correlated to unfavourable prognosis over a large range of cancers^5^. Currently, data are too limited to be able to investigate the prognostic role of ITH in MF. Some indirect evidence, such as the correlation between the presence of Pautrier microabscesses with the risk of progression^28^ and higher ITH in MF tumors as compared to early plaques^16^ suggest that this indeed may be the case. A more detailed understanding of the differences between functionally significant signalling pathways on the level of transcriptome and the protein is needed.

## Material and methods

### Sample collection, cryosectioning, laser capture microdissection (LCM) and sample preparation for whole-exome sequencing (WES)

Samples (4 mm punch biopsy and 10 ml of blood) were obtained from 7 patients after informed consent under ethics approval number HREBA.CC-16-0820-REN1 approved by Health Research Ethics Board of Alberta, Cancer Committee. All samples were collected and processed according to the ethics guidelines of Health Research Ethics Board of Alberta, Cancer Committee. Peripheral blood mononuclear cells (PBMC) were used as normal control except in sample MF18 where the epidermal cells were used as normal control for data analysis. Frozen biopsies were sectioned at 10 µm, transferred on 2 µm PEN membrane slides and stained with hematoxylin and eosin. Clusters of atypical cells representing malignant lymphocytes were microdissected from the dermis and the epidermis under 20 × or 40 × magnification in Leica DM6000B microscope (Wetzlar, Germany). The microdissected epidermal lymphocytes represented Pautrier microabscesses which could readily be identified based on their enlarged hyperchromatic nuclei, lighter cytoplasm and a cleavage separating them from the surrounding epidermis (Supplementary Fig. S2). Sequencing libraries were prepared with NEBNext Ultra II kit for Illumina (cat# E7645S) (New England Biolabs, MA) and exomes were captured with SSELXT Human All exon V6 + UTR probes (Agilent Technologies, CA). Samples were sequenced on Illumina HiSeq 1500 sequencer or NovaSeq 6000 platform. Detailed protocol for samples processing for storage and sequencing explained in previous methods^15^. The average depth for the samples is 168.66 × and for the controls is 167.51 ×. The read depth per sample is listed in Supplementary Table S2.

### Data analysis

To identify the TCR sequences, the fastq files were analyzed using MiXCR (version 2.10.0)^29^. To identify the genomic subclones, the raw sequences were mapped to hg38 genome assembly using BWA. The uBAM files are then corrected for duplicated and base quality score using MarkDuplicates and BaseRecalibrator respectively, which are packages part of GATK4 (version 4.0.10) best practices and Picard (2.18.14). Somatic variants (SVs) were identified by MuTect2 (version 2.1)^30,31^ and Strelka2 (version 2.9.10)^32^. Variants filtered as “Pass” from both variant callers were used for downstream analysis. Programs were used at their default setting. Variant effect predictor (VEP, version 95.2) was used to assign functional significance to the predicted SVs^33^. Titan-CNA (version 1.20.1) was used to identify copy number aberrations (CNA) and predict the tumour cell fraction (TCF)^33,34^. PhyloWGS (version 1.0-rc2) was used for phylogenetic analysis of the genetic subclones^33–35^.

TCR clonotypes were identified using MiXCR (version 2.10.0)^29^ analysis of the fastq files were analyzed to identify the TCR clonotypes, as described previously in detail^15,17^. Partial reads were filtered out as these might be the captures of only V or J sequences.The tcR^36^ package for R was used to calculate the overlapping clones. The number (n) of neoplastic TCRβ clonotypes was calculated to satisfy the equation

$$ \mathop \sum \limits_{i = 1}^{n} = TCRB_{i} \simeq TCF $$

where *TCRB*_*i*_ is the percentage of the TCRβ clonotype of *i-*rank (the rank *i* = 1 being the most abundant, dominant clonotype) and TCF is the tumor cell fraction in the sample calculated from WES using the Titan-CNA pipeline^17^. We assumed that the proportion of malignant T-cells cells with > 1 rearranged TCRB is negligible (allelic exclusion) and therefore the number of neoplastic TCRβ clonotypes *n* is equal to the number of malignant T-cell clones. We have previously established that TCR clonotypes obtained by MiXCR analysis of WES data were largely identical to those obtained using the traditional approach of PCR-amplification of CDR3 segments of rearranged TCR^15,37^.

## Supplementary information

Supplementary file 1

## Data Availability

The raw fastq files are submitted to the Sequence Read Archives (SRA) database under accession number PRJNA622539. The vcf files will be made available upon request to A.I.
