# Supplementary material for: Independent evolution of cutaneous lymphoma subclones in different microenvironments of the skin
Source: Sci Rep. 2020 Sep 23;10:15483. doi: 10.1038/s41598-020-72459-9 (PMC7511331; doi:10.1038/s41598-020-72459-9)
Supplement: Supplementary file 1 [file 41598_2020_72459_MOESM1_ESM.pdf]

Independent evolution of cutaneous lymphoma subclones in different microenvironments of the skin

Aishwarya Iyer[1,2], Dylan Hennessey[1,2], Sandra O'Keefe[1,2], Jordan Patterson[1], Weiwei Wang[5], Gane Ka-Shu Wong[1,4] and Robert Gniadecki[1-3,6*]

Supplementary figures and tables

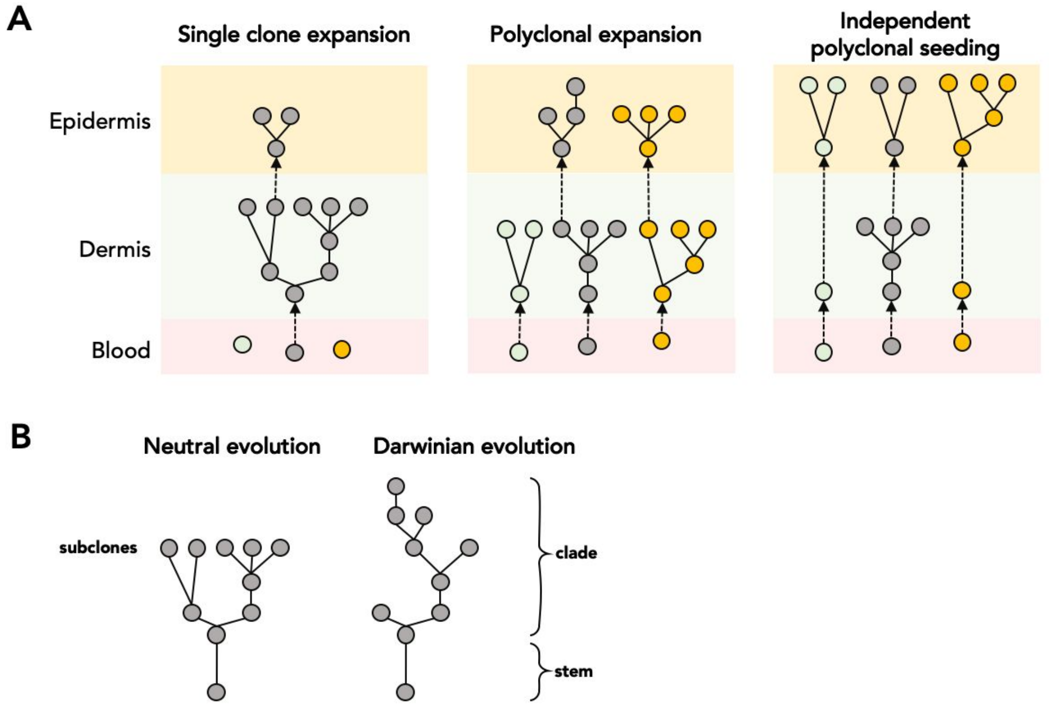

Figure S1: Possible scenarios of tumor evolution and generating intratumoral heterogeneity in MF. (A) Our previous work showed that lesions of MF are initiated by polyclonal circulating malignant T-cells homing to the skin where they undergo expansion and accumulation of mutations.[1],[2] Various clones (defined as T-cells sharing identical TCRβ clonotype) are highlighted by different colours. Expanding clones accumulate mutations and form subclones forming a phylogenetic structure. In lesions funded by a single T-cell clone (left), the entire lesion will comprise the same clonotype and the epidermal subclones will form a branch of the phylogenetic tree. If the lesion is initiated by diverse subclones (middle) that primarily proliferate in the dermis and secondarily infiltrate the epidermis the malignant cells in the epidermis and dermis would be polyclonal but epidermal malignant T-cells will form branches derived from dermal subclones. Finally, in the case of independent seeding of the dermal and epidermal niches (right), both compartments will harbour cells showing non-overlapping (or partially overlapping) clonotypes and independent patterns of mutational subclones. **(B)** Different shapes of phylogenetic tree characteristic for neutral evolution and

Darwinian evolution by natural selection. In neutral evolution, the shape of the phylogenetic tree is symmetrical (left) in contrast to Darwinian evolution where the extinction of the subclones will prune some branches (right). To avoid confusion we use the term “clone” as the group of T-cells of identical clonotype (i.e. sharing a common ancestry) rather than to mutationally identical groups of cells which we refer to as “subclones”. Clades are collections of several subclones.

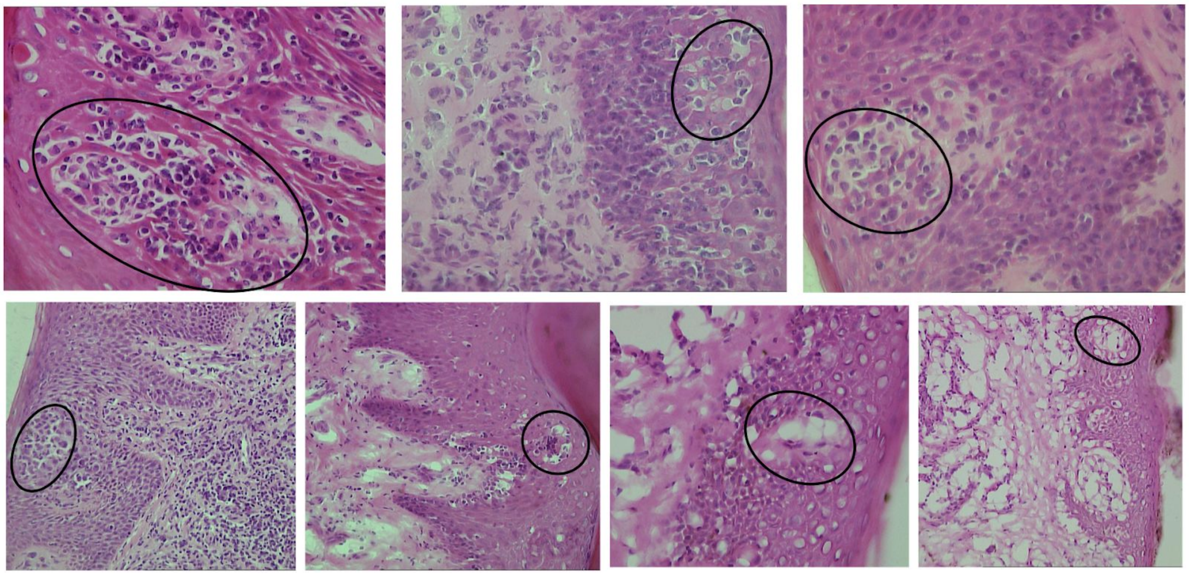

Figure S2: Histological identification of Pautrier microabscess in MF. Skin biopsies of MF lesions were sectioned at 10μ and stained with hematoxylin and eosin. Pautrier microabscesses are clearly discernible as clusters of atypical lymphoid cells with enlarged hyperchromatic nuclei in epidermis (black ovals)and separated from the surrounding keratinocytes by clefts, the artifacts created during cryosectioning. Representative images (magnification of 20x or 40x) of the H and E stained issues are presented for the 7 patients in the study.

Supplementary Table S1: Patient characteristics and samples included in the study

Patient ID (age [years], sex [M-male, F-female])	Sample ID	Lesion type	Diagnosis and stage
MF17 (70,M)	MF17E	Plaque	Mycosis Fungoides, IB
	MF17D		
MF18 (78, M)	MF18E	Plaque	Mycosis Fungoides, IB
	MF18D		
MF22 (56, F)	MF22E	Plaque	Mycosis Fungoides, IA
	MF22D		
MF23 (69, F)	MF23E	Plaque	Mycosis Fungoides, IA
	MF23D		
MF28 (65, M)	MF28E	Plaque	Mycosis Fungoides, IB
	MF28D		
MF41(77, F)	MF41E	Plaque	Mycosis Fungoides, IIB
	MF41D		
MF42 (82, M)	MF42E	Plaque	Mycosis Fungoides, IIIB
	MF42D		

Supplementary Table S2: Mean sample sequencing depth.

Samples	Sequences depth	Samples	Sequences depth
MF17D	108.213	MF22D	136.31
MF17E	154.403	MF22E	172.914
MF17PBMC	157.99	MF23PBMC	130.455
MF18 Control	121.632	MF23D	185.608
MF18D	117.821	MF23E	120.914
MF18E	159.641	MF28D	192.839
MF22PBMC	170.469	MF28E	191.769

Samples	Sequences depth	Samples	Sequences depth
MF28PBMC	122.576	MF42D	161.734
MF41D	156.038	MF42E	178.573
MF41E	124.5	MF42PBMC	123.09
MF41PBMC	146.391		

References for supplementary material

1. Iyer, A. *et al.* Skin colonization by circulating neoplastic clones in cutaneous T-cell lymphoma. *Blood* blood.2019002516 (2019) doi:10.1182/blood.2019002516.
2. Iyer, A. *et al.* Branched evolution and genomic intratumor heterogeneity in the pathogenesis of cutaneous T-cell lymphoma. doi:10.1101/804351.
